# A comprehensive overview of the relationship between RET gene and tumor occurrence

**DOI:** 10.3389/fonc.2023.1090757

**Published:** 2023-02-14

**Authors:** Lu Zhao, Na Wang, Dou Zhang, Yingjie Jia, Fanming Kong

**Affiliations:** ^1^ Department of Oncology, First Teaching Hospital of Tianjin University of Traditional Chinese Medicine, Tianjin, China; ^2^ National Clinical Research Center for Chinese Medicine Acupuncture and Moxibustion, Tianjin, China

**Keywords:** RET, thyroid cancer, lung cancer, breast cancer, targeted therapy

## Abstract

RET gene plays significant roles in the nervous system and many other tissues. Rearranged during transfection (RET) mutation is related to cell proliferation, invasion, and migration. Many invasive tumors (e.g., non-small cell lung cancer, thyroid cancer, and breast cancer) were found to have changes in RET. Recently, great efforts have been made against RET. Selpercatinib and pralsetinib, with encouraging efficacy, intracranial activity, and tolerability, were approved by the Food and Drug Administration (FDA) in 2020. The development of acquired resistance is inevitable, and a deeper exploration should be conducted. This article systematically reviewed RET gene and its biology as well as the oncogenic role in multiple cancers. Moreover, we also summarized recent advances in the treatment of RET and the mechanism of drug resistance.

## Introduction

Precision therapy changed the prospect of solid tumors. The intervention of aberrant tyrosine kinase has become the optimal target. Rearranged during transfection (RET) was first identified in 1985 in the transfection of NIH3T3 (1). RET gene has been confirmed to have a great role in the development of the kidney and nervous systems (2). The mechanism of RET aberrant activation was different from that of other receptor tyrosine kinases, which need both additional glial cell-derived neurotrophic factor (GDNF) family receptor-α (GFRα) and co-receptors (GFα1/2/3/4). The tripartite complex form (GDNF ligand+GFRα complex+RET kinase) stimulated RET by autophosphorylation and then triggered RAS, MAPK, ERK, PI3K, and AKT signaling pathways to promote tumor cell proliferation, migration, and differentiation (1, 3–5).

Many treatment advances have been made in recent years. Multikinase inhibitors including sunitinib, vandetanib, regorafenib, and alectinib were approved by the Food and Drug Administration (FDA) (6–10). However, their response rates were lower when compared with those of ALK or ROS1, and the off-target toxicity limited the application (11). Selective RET inhibitors LOXO-292 (selpercatinib) and BLU-667 (pralsetinib), which were approved by the FDA in 2020 with good clinical benefits and low incidence of serious adverse events, were more ideal (12, 13). It is worth mentioning that the two drugs have a strong intracerebral activity, which is in line with the carcinogenicity of RET. However, recent publications reported the novel acquired resistance to selpercatinib and pralsetinib (14). Second-generation RET inhibitors such as BOS172738, TPX-0046, TAS0953/HM06, and LOX-18228 are currently in clinical trials (15). Moreover, platinum-containing chemotherapy or immune-checkpoint inhibitors (ICIs) were also explored to increase the chances of drug resistance patients.

This article systematically reviewed RET gene and its biology as well as the oncogenic role in multiple cancers. Moreover, we also summarized recent advances in the treatment of RET and the mechanisms of drug resistance. Finally, we analyzed the opportunities and challenges and, then, gave proposals for this portion of patients to maximize their survival time in the future.

## Function and biology of RET

RET gene was first identified in 1985 from the transfection of NIH3T3 (16). It was located in chromosome 10 (10q11.2) and contained 21 exons, its full length was 60 kb, and it was the receptor for GDNF (17). In addition to GDNF, this family also included artemin (ARTN), neurturin (NRTN), and persephin (PSPN). RET gene was required for the development of the brain and nervous systems, thyroid and lung tissues, and others (18). Unlike other RTKs, RET gene was not bound directly to the ligands. Instead, the RET ligands first bind to the GFRα receptor. The GFL–GFRα complex then mediated RET homodimerization, which lead to autophosphorylation and then activated the proliferation pathways such as MAPK, PI3K, JAK-STAT, PKA, and PKC (19, 20).

PI3K-AKT-mTOR and RAS-RAF-MEK-ERK were the major ways of cell survival, proliferation, migration, and differentiation (21). Three general mechanisms of aberrant RET activation will trigger the above pathways: in-frame RET fusions, targeted mutation, and aberrant overexpression (22, 23). However, the different sites lead to a different degree of tyrosine kinase transformation. Three main ways were included: sudden changes of codons in the extracellular region result in the transform of cysteine residues, codon mutations in the transmembrane region cause two receptor proteins to draw nearby non-covalent bond, and ATP binds to its site easily made by codon mutations in the intracellular regions (24–26). Among them, RET point mutation frequently occurred in multiple endocrine neoplasias and medullary thyroid carcinoma (27). However, RET fusion has been commonly reported in papillary thyroid and non-small cell lung cancers (14, 28).

## RET gene and tumor occurrence

### RET expression in lung cancer

Lung cancer is the most prevalent malignant tumor in the world with a poor survival rate and faster progression. Non-small cell lung cancer (NSCLC) accounted for 80% to 85% (29, 30). For patients who are not eligible for targeted therapy, platinum-based chemotherapy is the standard treatment. However, their survival time is less than 12 months. RET fusion was discovered in approximately 1%–2% of NSCLC (31). KIF5B was the most common type in RET fusion, and 47 other partners have been identified so far (7, 32). The clinical and pathological features of RET fusion NSCLC patients differ from those caused by other oncogenes. RET fusion NSCLC patients correlated with adenocarcinoma histology, never-smoking status, younger age, more advanced stage disease, and potentially higher chemo-sensitivity (pemetrexed-based regimens) (33, 34). It is of high concern that RET fusion NSCLC patients are more likely to have brain metastases, and the incidence is up to 27% (35, 36). Therefore, developing novel agents with blood–brain barrier (BBB) permeability is necessary. Multikinase inhibitors (MKIs) showed inferior activity in RET-NSCLC, compared with EGFR or ALK. The off-target toxicity and suboptimal intracerebral activity also limited its application in clinical. ICIs in driver gene mutation tumors are controversial. The present studies reported disappointing efficacy with ICI monotherapy in this portion of patients. However, ICI-based combination therapy may bring hope in the future (37, 38). Thus, chemotherapy remained a reasonable choice until RET-selective tyrosine kinase inhibitors (TKIs) emerged.

### RET expression in thyroid cancer

Thyroid cancer only accounts for 3%–4% of all human cancers commonly caused by ionization radiation (39). Nevertheless, it is prevalent in endocrine neoplasia with the highest increase in the past two decades. Thyroid cancer is categorized into four different types [papillary thyroid carcinoma (PTC), follicular thyroid carcinoma (FTC), anaplastic thyroid cancer (ATC), and medullary thyroid cancer (MTC)]. RET mutations were most commonly found in PTC and MTC (40). Indeed, the activation mechanisms were different in the two types. Chromosomal rearrangements were found in PTC, and somatic mutations lead to MTC (41).

PTC accounts for 85% of thyroid cancer and is the first human cancer associated with RET fusion. RET/PTC chimeric protein formed dimers that are required for oncogenic activation, which activated the RAS/MAPK/ERK pathway to promote proliferation and migration. An assay that targeted 244 cancer-related genes detected RET fusion in 4.35% PTC. Subsequently, in The Cancer Genome Atlas (TCGA) study, which enrolled 500 PTC patients, 6.8% had RET fusion. The high-frequency forms were CCDC6-RET (RET/PTC1) and NCOA4-RET (RET/PTC3), which were the consequences of double-stranded breaks caused by ionizing radiation (42). RET fusion has been reported more commonly in pediatrics and dose-dependently with irradiation. The Chernobyl accident remained an example that activated the MAPK pathways by chromosomal rearrangement. Approximately 58% aged <10 years old patients harbored RET fusion, and 50% of PTC patients who were exposed to high radiation doses (>0.5 Gy) had RET fusion (41, 43). Regrettably, the relationship between RET rearrangement and the prognosis of PTC is still controversial. Some studies confirmed that RET/PTC is a more aggressive phenotype combined with advanced-stage disease. On the contrary, other trials hold that there was no significant correlation between RET/PTC and tumor aggressiveness (14, 39, 44).

In contrast to PTC, MTC is a rare type (2%–4%) of thyroid cancer. Radiation exposure is not associated with RET fusion in MTC compared to PTC (45). MTC included five subtypes, but MEN2A and MEN2B are the most common. The current research indicated that MEN2A is related to the RET-C634 mutation (46). Mulligan analyzed 118 unrelated families and found that the RET-C634 mutation occurred in 95% of MEN2A families (47). In agreement with their earlier study, they did not detect the RET-C634 mutation in MEN2B families. Instead, MEN2B always shared the RET-M918T mutation with a 95% detection rate. It was first detected in 1994 by a separate study that detected the mutation in 34 unrelated MEN2B patients (48). Moreover, many studies have confirmed that RET mutations in MEN2A and MEN2B were reliable biomarkers for the identification of highly aggressive MTC.

### RET expression in breast cancer

Breast cancer (BC) is the most common cancer in women, with approximately 1.7 million people diagnosed every year, and RET alteration occurrence is approximately 1.2% (49). RET amplification is the most common, followed by RET fusion. Most RET mutations in BC appear after drug resistance, and CCDC6-RET and NCOA4-RET occur frequently (50). RET is actionable in ER^+^ BC. Gattelli identified that RET activation promoted proliferation and migration in ER^+^ BC patients (8). Plaza-Menacho showed that RET modulates the sensitivity of ER^+^ BC to endocrine therapy and that activated RET promotes estrogen-independent activation of ERa, which suggested an interference between RET and the ERa pathway in endocrine-resistant BC (51). After that, Isacke also determined that RET signaling was hyperactivated in aromatase-resistant ER^+^ BC (52). Although RET expression was related to ER in luminal BC, it lacked prognostic significance as an independent biomarker. In addition to ER^+^ BC, RET was also actionable in HER2-enriched and triple-negative BC patients who failed targeted therapy (53). A recent study showed that trastuzumab resistance was associated with the activation of the RET–HER2 signaling axis (54).

### RET expression in other tumors

RET mutations also occurred in other tumors. For example, G533C was confirmed to increase proliferation and migration in colon cancer (55). In pancreatic cancer, RET led to lymphatic invasion and was upregulated in ductal adenocarcinoma (56). In kidney cancer, RET was confirmed to predict survival and the high expression results in a shorter survival time. In prostate cancer, moderately to poorly differentiated tumors displayed overexpression of RET (57). In summary, RET was increasingly recognized as an oncogene and a potential target in multiple tumors.

## RET kinase inhibitors

### Non-selective MKI

Multi-targeted drugs are being used in RET mutation cancers in the early stage. For example, type I inhibitors such as vandetanib and lenvatinib were confirmed to bind to the ATP in an active conformation of RET kinase. Famous type II inhibitors such as cabozantinib and sorafenib can bind to the ATP in an inactive conformation. However, the clinical benefits (lower overall response rate (ORR) and shorter progression-free survival (PFS)) and significant off-target toxicities limited their application. A phase II clinical trial (NCT01639508) reported that cabozantinib had not reached the endpoints with 28% ORR, 5.5 months median PFS (mPFS), and 9.9 months median overall survival (mOS) (7). Similarly, a phase III clinical trial (NCT00704730) in MTC harboring RET-M918T showed that OS was 6.6 months (58). Also, lenvatinib (NCT01877083) in RET fusion NSCLC yielded a relatively low response (ORR = 16%, mPFS = 7.3 months) (59). Subsequently, a clinical study by Carlomagno identified that vandetanib may not yield clinical efficacy. The ORR was 18%, mPFS was 4.5 months, and mOS was 11.6 months in RET fusion NSCLC (6). After that, randomized, phase III, registrational trials confirmed that cabozantinib and vandetanib in patients with advanced MTC were also unspectacular, the ORR was 28% and 45%, and mPFS was 7.2 and 11.2 months, respectively, and uncontrollable adverse events frequently occurred (60). To sum up all the above studies, MKIs were not outstanding agents for RET mutation patients.

### RET selective TKI

BLU-667 (pralsetinib) and LOXO-292 (selpercatinib) were two highly selective RET inhibitors with good efficacy and tolerable adverse effects. Currently, the clinical data of the two drugs have been recently released. The ensuing drug resistance has become a new challenge. Other RET inhibitors such as BOS172738, GSK3352589, and GSK3179106 are currently undergoing phase I clinical trials.

Selpercatinib is an oral RET inhibitor designed to overcome the weaknesses of MKIs. The *in vitro* and *in vivo* studies revealed that selpercatinib can inhibit wild and altered RET, meanwhile holding back KDR/VEGFR2 activity. LIBRETTO-001, a global phase I/II trial, demonstrated that selpercatinib had the perfect outcomes in RET fusion NSCLC patients with a 68% ORR. The ORR of the brain metastases patients also reached 91%. The median diagnostic odds ratio (mDOR) was 20.3 months, and mPFS was up to 18.4 months (13). After that, LIBRETTO-321 was performed to evaluate the efficacy and safety of selpercatinib in Chinese patients. Consistent with the previous conclusions, the ORR was 61.1%, and 90% of the patients remained in continuous remission after 6 months (28). As for RET-mutant MTC, LIBRETTO-001 showed a 56% ORR, and the ORR was similar regardless of whether MKI has been used before (61). Therefore, the FDA accelerated the approval of selpercatinib for RET mutation NSCLC and MTC patients in 2020. Currently, LIBRETTO-121 and LIBRETTO-431 are ongoing to confirm the effectiveness of selpercatinib in other tumors.

Same as selpercatinib, pralsetinib is also an ATP-competitive inhibitor that selectively inhibits RET. ARROW was a single-arm phase I/II trial that demonstrated that 90% of PTC and MTC have radiographic tumor reduction with pralsetinib. The ORR was 60% (disease control rate (DCR) 100%) *vs.* 63% (DCR 94%) in RET fusion NSCLC and RET mutation MTC. Nine patients with brain metastases showed an intracranial response rate of 56% in MTC (12). Remarkably, pralsetinib can be widely used regardless of RET fusion partner (62). Based on these data, pralsetinib was granted by the FDA in 2020 for RET mutation NSCLC and MTC. Subsequently, China’s State Food and Drug Administration [National Medical Products Administration (NMPA)] also approved pralsetinib for Chinese patients in March 2021. Other ongoing clinical trials such as NCT04222972, NCT04222972, and NCT04760288 were aimed to assess the application of pralsetinib in other tumors.

## Other therapy

ICIs have become the keystone in cancer treatment and are considered a salvage treatment for patients with actionable driver alterations after targeted therapies. Building on previous experience that ICI monotherapy was unsatisfactory, combination therapy has been increasingly applied in RET mutation cancers. A clinical trial showed that bevacizumab+carboplatin+pemetrexed can highly prolong the survival time of RET fusion NSCLC patients. The mPFS was 6.6 months *vs.* 5.7 months (63). Subsequently, Guisier also determined the effectiveness of ICIs-based combination therapy for RET mutation cancers in a real-world setting. Among 107 patients, only nine had RET translocation. Before ICIs, they had received at least one line of treatment. The results showed the mPFS was 7.6 months, the median DOR was 4.7 months, and the ORR was 38% (64). A multicenter retrospective study reported a high DCR (60%) in RET mutation patients who failed targeted therapy, with a conclusion that was in line with that of another trial (ORR = 58%, mPFS = 5.4 months, mOS = 19 months) (65). Based on the above data, the therapeutic value of ICIs in patients with RET mutation has become clear. After targeted therapy, ICIs or in combination with chemotherapy will bring new vitality to this portion of patients.

Given that those RET selective inhibitors were recognized recently, the majority of patients are still being treated with chemotherapy. Platinum-doublet chemotherapy is the standard regimen in RET mutation NSCLC patients (66). A multicenter retrospective study showed that 65 RET fusion NSCLC patients used platinum-based chemotherapy as the first-line treatment, the ORR was 51%, and the mPFS was 7.8 months. Another global trial also showed optimistic results (mPFS = 6.6 *vs.* 7.8 months, OS = 23.6 *vs.* 24.8 months) in RET mutation NSCLC and MTC patients (67, 68).

## The mechanistic pathway of drug resistance

According to the present data, both instinct and acquired resistance of RET TKIs exist. Understanding the mechanistic pathway of targeted agents is imperative to prolong remissions due to the drugs. On-target and off-target resistance were included to explain the underlying mechanisms of drug resistance.

### On-target mechanism of drug resistance

There was an acquired resistance inside the target kinase, which was continuously activated by kinase inhibitors. Gatekeeper mutations and solvent front mutations are two common types in MKI and TKI (15). The construction of the *in vitro* model confirmed that RET V840 gatekeeper mutations mediated drug resistance in the following two ways: lead the spatial conflict between leucine and methionine side chains and the 4-bromo-2-fluorophenyl group and increase the adenosine triphosphate affinity (22). A recent study showed that a novel RET inhibitor, SYHA1815, can overcome this resistance, which may be a new direction for drug development (69). However, Gly-810 is representative of solvent front mutation, which is located at the solvent front of the ATP binding pocket (70). Solomon reported that RET G810R, G810S, and G810C mutations occurred in three NSCLC cases with RET fusion after selpercatinib (71). Of note, their study also described that RET V840 gatekeeper mutation and G810 solvent front mutation could be present at the same time. Therefore, more clinical studies need to explore whether RET-selective TKI was able to prevent gatekeeper mutation.

### Off-target mechanism of drug resistance

Off-target resistance activates different intracellular pathways that bypass the kinase-mediated signal. MET, EGFR, BRAF, and RAS were all reported in recent trials, of which MET was common as a recurring and potential type of resistance of selpercatinib and pralsetinib (72). A retrospective clinical trial analyzed 20 RET fusion NSCLC patients who were resistant to selpercatinib and pralsetinib, and they found 15% MET amplification and 10% G810C/S mutation. EGFR would activate downstream pathways and disrupt the combination of kinase inhibitors to restore fusion signaling complexes, which promote proliferation and hide RET inhibitors (73, 74). RAS and BRAF mutations were reported in two and one KIF5B-RET fusion NSCLC patients, respectively, who received selpercatinib, yet more experimental validation is still needed.

### Next-generation RET inhibitors

While not overwhelmingly dominant, RET resistance mutations are recurrent in patients treated with selpercatinib or pralsetinib. For these patients, novel RET inhibitors harboring potency against the resistance mutations are needed. Next-generation RET inhibitors including BOS172738, TPX-0046, TAS0953/HM06, and LOX-18228 were designed to solve the above problems. BOS172738 could overcome RET-G810 resistance and showed good activity in patients with RET fusion tumors (55). A phase I clinical trial showed good efficacy and safety with 33% ORR. TPX-0046 presents perfect benefits *in vitro* and *in vivo* RET fusion cancers. It can overcome RET-G810 resistance. A phase I/II clinical trial (NCT04161391) is ongoing to evaluate the efficacy and safety of TPX-0046 in advanced cancers harboring RET mutants (15). HM06, another selective RET inhibitor, circumvents RET-V804X gatekeeper mutation and RET-G810X resistance mutations. This drug is currently in phase I/II clinical trials (NCT04683250) in a patient with RET mutation (75). Lastly, LOX-18228 can inhibit RET-V804X and RET-G810X mutations, which have a promising use after first-generation RET inhibitors. LOX-18228 is now entering phase I clinical trials.

## Summary and prospects

RET proto-oncogene was identified more than 30 years ago, and the rearrangement and mutation of RET have been reported in a variety of cancers, including thyroid cancer, non-small cell lung cancer, and breast cancer. Currently, targeted therapy and immune checkpoint inhibitors brought new life to this portion of patients. Multiple kinase inhibitors easily generate toxicity because of the off-target effects. Most of them were not authorized by the FDA (76–80). Immune checkpoint inhibitors as the post-line treatment option were sensible for driver gene mutation patients (38, 63, 81, 82). The highly selective RET inhibitors such as selpercatinib and pralsetinib provided considerable benefit in both MTC and NSCLC patients and were authorized for the first-line treatment (83, 84). However, like other inhibitors, on-target or bypass resistance of RET-TKI will become more common. Several novel RET inhibitors, which cover not only the drug-resistant site but also other RTKs that can activate parallel signaling pathways, are at an early stage (26, 85). Of note, further research still needs to explore the broader coverage of potential resistance mechanisms, and combination therapies to optimally pathways are also important.

## Author contributions

LZ and FK contribute equally to this article. All authors contributed to the article and approved the submitted version.
