# Supplementary material for: A comprehensive overview of the relationship between RET gene and tumor occurrence
Source: Front Oncol. 2023 Feb 14;13:1090757. doi: 10.3389/fonc.2023.1090757 (PMC9971812; doi:10.3389/fonc.2023.1090757)
Supplement: Supplementary file 1 [file Table_1.docx]

**Table Clinical trials of RET-targeting inhibitors**

| **Classification** | **Drug** | **NCT Number** | **Trial Group** | **Trial Design** | **Primary Endpoints** | **Secondary Endpoints** |
| --- | --- | --- | --- | --- | --- | --- |
| **Multi-kinase inhibitor** | Cabozantinib | NCT01639508 | 26 NSCLC patients harboured a RET rearrangement | Patients received cabozantinib 60mg orally in 28-day cycles | ORR:28% | mPFS:5.5months  mOS:9.9months |
|  |  | NCT00704730 | 219 MTC patients with RET mutation | Patients were randomized 2:1 to receive either a single oral  daily dose of 140 mg cabozantinib or a placebo in 4-week cycles | PFS:14months | OS:NA  ORR:32%  DOR:NA |
|  | Vandetanib | NCT01823068 | 18 NSCLC patients harboured a RET rearrangement | Patients began on once daily vandetanib at 300 mg with one cycle of 4 weeks | ORR:18% | PFS:4.5months  DCR:65%  OS:11.6months |
|  | Sorafenib | NA | 16 MTC patients with RET mutation | Sorafenib was administered at the dose of 400 mg orally twice daily on a  continuous basis | ORR:NA | NR |
|  | Alectinib | NCT03194893 | 4 NSCLC patients harboured a RET rearrangement | Patients recieved alectinib 600 mg twice a day (BID) orally twice daily | Safety/efficacy  endpoints; DFS | NR |
| **First-generation of**  **RET inhibitor** | Pralsetinib | NCT03037385 | 589 patients with RET-altered MTC, NSCLC and other solid tumors | The study consists of 2 parts  **Phase1**:paralsetinib30-600mg(PO QD or BID)  **Phase2**:400mg(QD) | **NSCLC and MTC**  ORR:53% *vs* 33% | **NSCLC and MTC**  DCR:79% *vs* 51% |
|  |  | NCT04760288 | 198 MCT patients with RET mutation | Patients received pralsetinib at a dose of 400 mg, as per the dosing schedule described above | PFS:NA | TTF:NA  ORR:NA  OS:NA |
|  |  | NCT04222972 | 281 patients with RET fusion NSCLC | Pralsetinib administered orally | PFS:16.5months | ORR:72%  OS:NA  DOR:22.3monts |
|  | Selpercatinib | NCT03157128 | 253 patients with RET fusion-positive NSCLC | Selpercatinib, 160 mg orally twice daily(28day cycles)  **Phase1**:Multiple doses of selpercatinib  **Phase2**:The maximum tolerated dose | Phase I: MTD,  RP2D (258)  Phase II: ORR | PFS:16months  OS:NA  DOR:17months  ORR:64% |
|  |  | NCT04280081 | 77 Chinese patients with RET-altered  thyroid cancers | Selpercatinib 160 milligrams (mg) administered orally twice daily (BID) | ORR:57.7% | DOR:NR |
|  |  | NCT04194944 | 250 NSCLC patients harboured a RET rearrangement | Selpercatinib administered orally | PFS:NA | DOR:17.5months  ORR:85%  OS:NA |
| **Next-generation of**  **RET inhibitor** | TPX-0046 | NCT04161391 | 462 patients with advanced or metastatic solid tumors harboring RET mutations or alterations | **Phase1**:Dose escalation and dose Expansion  **Phase2**:Efficacy evaluation | DLT, RP2D,  ORR | ORR  DOR  OS |
|  | HM06 | NCT04683250 | 202 participants with advanced solid tumors with RET gene abnormalities | **Phase1:**Dose escalation and dose expansion  **Phase2**:at recommended Phase 2 dose in three different populations | Phase I: MTD  Phase II: ORR | ORR  DCR  PFS  OS  DOR |
