# Supplementary figures and images for: A comprehensive overview of the relationship between RET gene and tumor occurrence
Source: Front Oncol. 2023 Feb 14;13:1090757. doi: 10.3389/fonc.2023.1090757 (PMC9971812; doi:10.3389/fonc.2023.1090757)

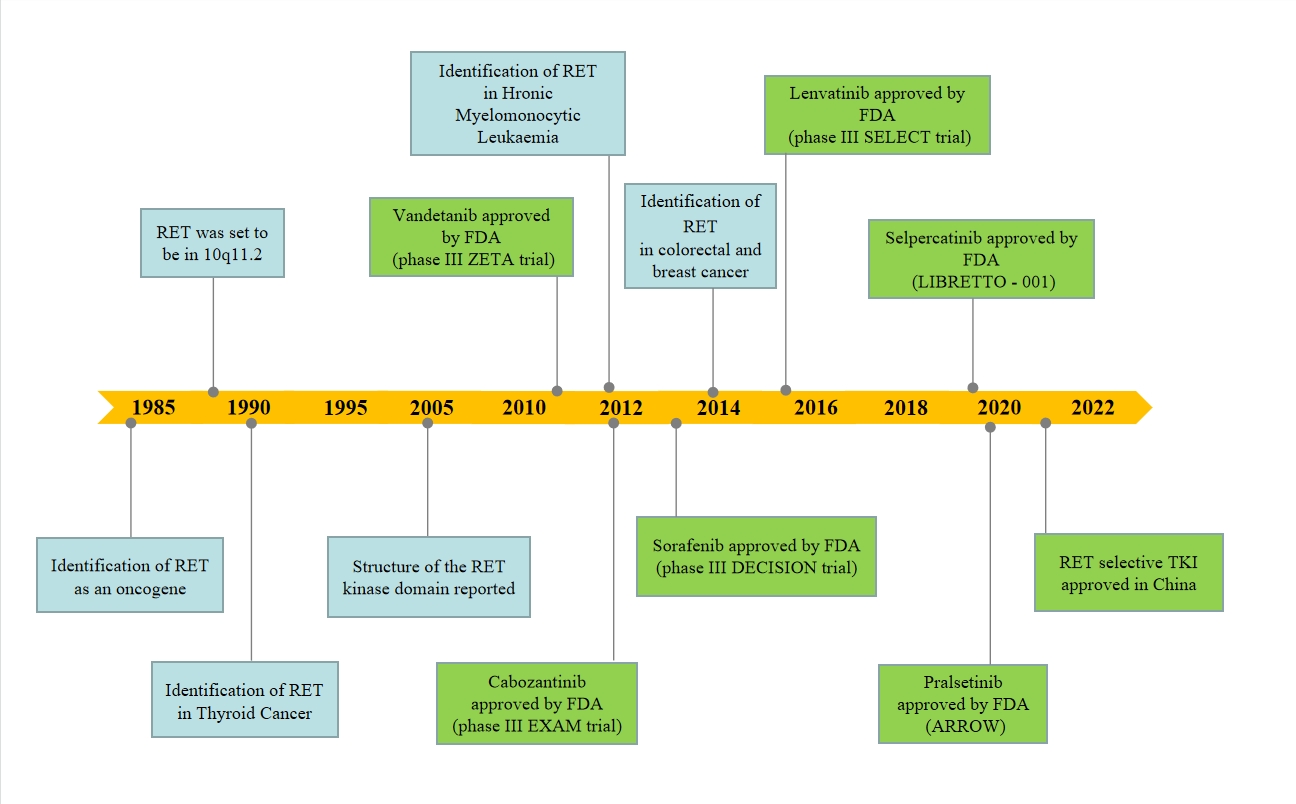

Supplement: Supplementary file 2 [file Image_1.jpeg]

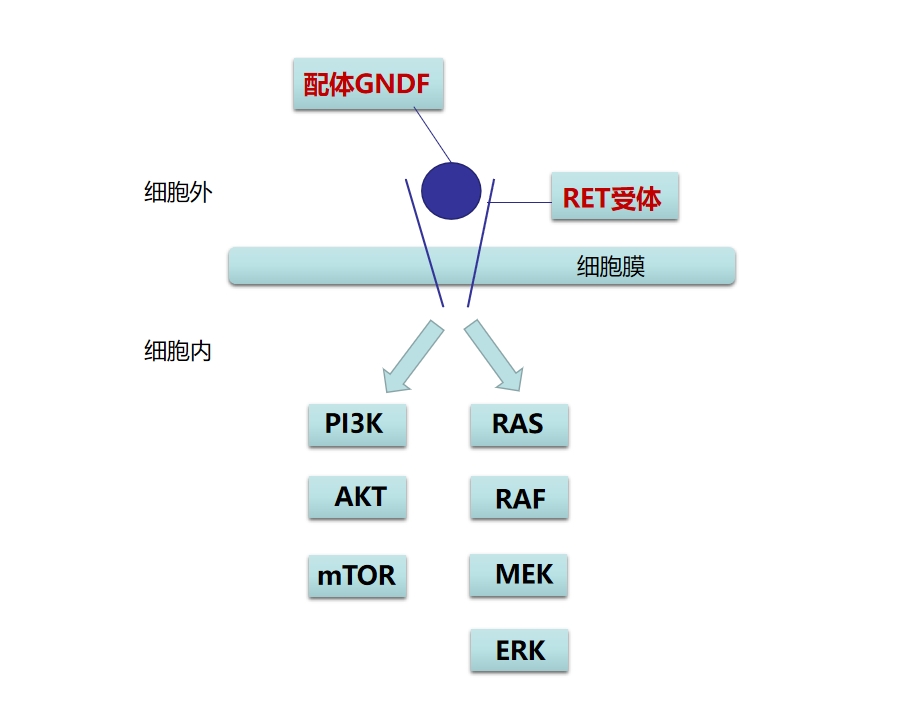

Supplement: Supplementary file 3 [file Image_2.jpeg]

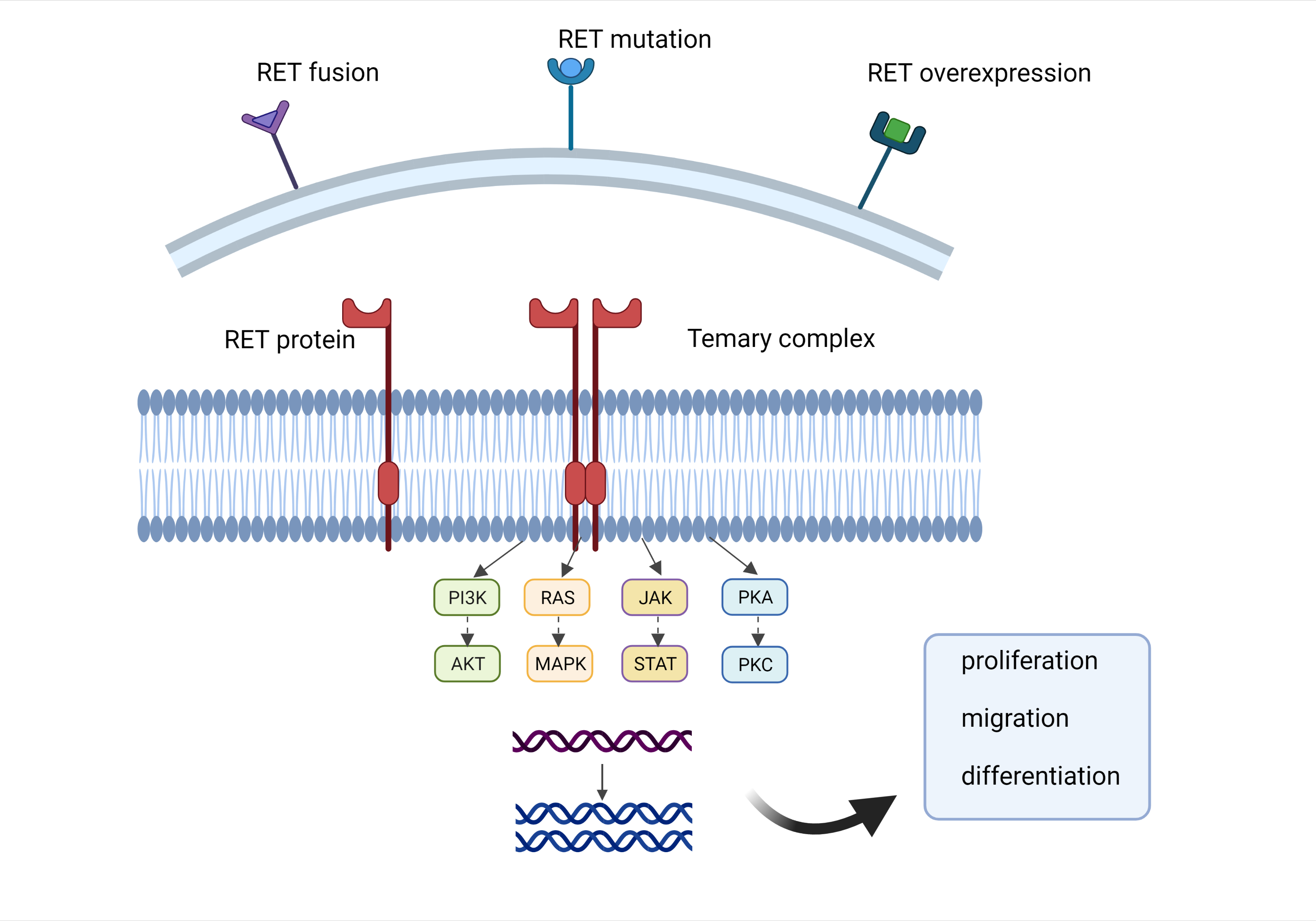

Supplement: Supplementary file 4 [file Image_3.jpeg]
